# Ginkgo Biloba Extract EGB761 Ameliorates the Extracellular Matrix Accumulation and Mesenchymal Transformation of Renal Tubules in Diabetic Kidney Disease by Inhibiting Endoplasmic Reticulum Stress

**DOI:** 10.1155/2021/6657206

**Published:** 2021-03-23

**Authors:** Jiarui Han, Xinxin Pang, Xiujie Shi, Yage Zhang, Zining Peng, Yufeng Xing

**Affiliations:** ^1^Henan University of Chinese Medicine, Zhengzhou, 450046 Henan, China; ^2^Department of Nephropathy, Henan Provincial Hospital of Traditional Chinese Medicine/The Second Hospital Affiliated to Henan University of Chinese Medicine, Zhengzhou, 450002 Henan, China

## Abstract

The study is aimed at investigating the effects of Ginkgo biloba extract EGB761 on renal tubular damage and endoplasmic reticulum stress (ERS) in diabetic kidney disease (DKD). A total of 50 C57BL/6 N mice were randomly divided into the normal group, DKD group, DKD+EGB761 group (36 mg/kg), and DKD+4-phenylbutyrate (4-PBA) group (1 g/kg). The DKD model was replicated by high-fat diet combined with intraperitoneal injection of streptozotocin (STZ). Renal tubular epithelial cells (HK-2) were divided into the control group, high-glucose group (30 mmol/L), EGB761 group (40 mg/L, 20 mg/L, 10 mg/L), TM group, and TM+4-PBA group. After 8 weeks of administration, expressions of serum creatinine (Scr), blood urea nitrogen (BUN), 24 h urinary protein (24 h Pro), fasting blood glucose (FBG), *β*_2_-microglobulin (*β*_2_-MG), and retinol binding protein 4 (RBP4) of mice were tested. The pathological changes of renal tissue were observed. The expressions of extracellular matrix (ECM) accumulation and epithelial-mesenchymal transition (EMT) markers *α*-smooth muscle actin (*α*-SMA), E-cadherin, fibronectin, and collagen IV, as well as the ERS markers GRP78 and ATF6, were tested by Western blot, qPCR, immunohistochemistry, or immunofluorescence. EGB761 could decrease the Scr, BUN, 24 h Pro, and FBG levels in the DKD group, alleviate renal pathological injury, decrease urine *β*_2_-MG, RBP4 levels, and decrease the expression of *α*-SMA, collagen IV, fibronectin, and GRP78, as well as ATF6, while increase the expression of E-cadherin. These findings demonstrate that EGB761 can improve renal function, reduce tubular injury, and ameliorate ECM accumulation and EMT in DKD kidney tubules, and the mechanism may be related to the inhibition of ERS.

## 1. Introduction

Diabetic kidney disease (DKD) is one of the major microvascular complications of diabetic patients and has become the major causes of end-stage renal disease (ESRD) worldwide [1]. The etiology of DKD is complex [2, 3], including genetic factors, glycolipid metabolism disorder, inflammatory response, oxidative stress, autophagy, endoplasmic reticulum stress, and immune response. It is pathologically manifested as extracellular matrix (ECM) deposition and glomerular basement membrane thickening and eventually develops into tubulointerstitial fibrosis and glomerulosclerosis. Clinical treatment like adequate hypoglycemia, hypotension, and application of RAS blockers, however, still cannot effectively prevent its' progression to ESRD. Therefore, further investigation is needed to explore the pathogenesis of DKD and the mechanism of potential other interventions [4].

In recent years, renal tubular injury has attracted more attention in the progression of DKD. Renal tubular tubulointerstitial fibrosis (RTF) is a common outcome with the progressive development of almost all chronic kidney diseases [5] and is an important pathological basis for ESRD. ECM deposition and epithelial-mesenchymal transition (EMT) play an important role in the development of RTF. In DKD, the long-term infiltration of inflammatory mediators and high glucose stimulation results in renal tubular epithelial cells undergoing inflammatory changes, and the transcription of various ECM proteins was activated, causing excessive ECM accumulation in renal tubular interstitium [6, 7] and consequent renal tubular interstitial fibrosis. EMT is a progress that renal tubular epithelial cells transform into mesenchymal cell. Due to the presence of high glucose, urine proteins, oxidative stress, and renal tubular epithelial cells transform into myofibroblasts to resist to the environmental damage, then enters in interstitium and abnormally synthesizes ECM [8, 9], thus aggravating renal tubular interstitial fibrosis. Therefore, interfering with ECM accumulation and EMT in renal tubular epithelial cell is of great significance in DKD treatment.

Ginkgo biloba extract is an active compound in the traditional Chinese medicine extracted from the plant Ginkgo biloba. EGB761 is the standardized extract of Ginkgo biloba produced by the Schwabe company in Germany, and its main active component is flavonoids and terpenoids. It is widely used in clinical practice and well tolerated. Previous studies showed that Ginkgo biloba extract has a definite curative effect on DKD [10–12], but its mechanism of action still needs to be fully clarified. Here, we explored the effects of EGB761 on ECM accumulation and EMT in DKD mice and renal tubular epithelial cells (HK-2), as well as its mechanism. Our results provided evidence on the protective effect and mechanism of EGB761 against DKD.

## 2. Materials and Methods

### 2.1. Animals

Fifty of C57BL/6 N male mice were fed freely with food and water. The animals had a day and night cycle of 12-hour, with a 22-25°C room temperature. They were fed with normal diet for one week. The study was approved by the Animal Ethics Committee of the Henan University of Chinese Medicine.

### 2.2. Animal Treatment and Grouping

Ten mice were selected as the control group, and the remaining 40 mice were prepared for DKD model. To establish the DKD model, the mice were firstly given high-fat diet for 4 weeks, then fasted for 10 hours, and intraperitoneally injected with STZ (S0130, Sigma Company, USA) solution (mixed with pH 4.5 sodium citrate buffer) at the dose of 50 mg·kg^−1^ for 5 days, while the control group was given normal diet, and the same amount of sodium citrate buffer was injected intraperitoneally. One week later, the random blood glucose measured by the tail − cutting method ≥ 13.9 mmol/L was regarded as the successful replication of the diabetic model. The diabetic model mice continued to be given high-fat diet; then, the urine protein was detected 8 weeks later. The positive mice were then divided into the DKD group, DKD+EGB761 group, and DKD+4-phenylbutyrate (4-PBA) group. According to the equivalent metrology of human and animal, the DKD+EGB761 group was given EGB761 (2280219, Dr Willmar Schwabe, Germany) 36 mg/kg, and the DKD+4-PBA group was given 4-PBA, a classic inhibitor of endoplasmic reticulum stress (ERS), (SML0309, Sigma Company, USA) dissolved in NaOH solution (50 g·L^−1^, 1 g·kg^−1^). The control group and DKD group were given the same volume of normal saline. After 8 weeks of administration, eyeballs were removed to take blood, and kidneys were taken to detect various indexes.

### 2.3. Biochemical Index Detection

During the administration of the drug, fasting blood glucose (FBG) and 24-hour urinary protein (24 h Pro) were detected every two weeks; After the last administration, the mice were deprived of food but not water for 24 hours. Remove the eyeballs to collect blood and take the kidneys, serum creatinine (Scr), and blood urea nitrogen (BUN). FBG was tested by tail tip blood (OneTouch Ultra Glucometer, Johnson Co, LTD, USA), Scr (C011-2), BUN (20160511) ,and 24 h Pro (C035-2) that were detected by the kit (Nanjing Jiancheng Institute of Biological Engineering, China).

### 2.4. Hematoxylin-Eosin (H&E), Periodic Acid–Schiff (PAS), and Masson's Staining

Part of the renal tissue was taken, firstly fixed by 4% paraformaldehyde, then embedded in paraffin wax, and 4 *μ*m tissue was sliced, then dewaxed, and washed with H&E, PAS, and Masson's staining, and finally, light microscope was used to observe the pathological changes.

### 2.5. Transmission Electron Microscope

Part of the renal tissue was taken, firstly fixed with 2.5% glutaraldehyde, dehydrated, embedded, and sliced, and the ultrastructure of glomeruli and tubules was observed by transmission electron microscope.

### 2.6. Immunohistochemistry

The renal tissue was taken, firstly fixed in 4% paraformaldehyde, and embedded in paraffin wax. After dewaxed, endogenous peroxidase was blocked by 3% H_2_O_2_, microwave oven was used to repair surface antigen, goat serum was used for blocking surface antigen, and appropriate primary antibodies GRP78 (1 : 200) and ATF6 (1 : 200) were added, respectively, and incubated at 4°C overnight. After reheating, secondary antibody was added. After incubation at 37°C for 30 minutes, DAB color developing solution was added. Hematoxylin was added and dehydrated step by step, then observed under the microscope.

### 2.7. ELISA

After the last administration, the urine of the mice was collected, and the levels of *β*_2_-microglobulin (*β*_2_-MG) (KGE019, R&D Corporation, USA) and retinol binding protein 4 (RBP4) (DRB400, R&D Corporation, USA) were detected.

### 2.8. Cell Culture

HK-2 cells were purchased from Saibaikang Cell Bank (ATCC, China), cultured in DMEM (0034317, Hyclone, USA), which supplemented with 10% FBS (Invitrogen, Carlsbad, CA, USA) and 100 units/mL penicillin-streptomycin. HK-2 cells were i ncubated at 37°C, with 95% air and 5% CO_2_, subcultured every 3-4 days. The culture medium was replaced every 2-3 days.

### 2.9. Cell Treatment

Seeded for 2 or 3 days, serum-free medium was added and incubated for further 24 h. Then, high glucose (30 mmol/L) or high glucose combined with EGB761 at different concentrations (10, 20 or 40 mg/L) was added for further 48 h. Besides, TM, a classic activator of ERS (654380, Sigma) at 5 ug/mL or 4-PBA at 1 mmol/L, was added for 48 h before the treatment with EGB761 (20 mg/L). TM was used as ERS-positive control, while high glucose as positive control of ECM deposition and EMT.

### 2.10. Real-Time Quantitative PCR

TRIzol reagent (Thermo Fisher Scientific, Shanghai, China) was used to extract total RNA. Transcript Revers Transcription Kit (KIT0305, Applied Biosystems) was used to get single-stranded cDNA. SYBR Green Realtime PCR Master Mix (E090, SinoBio) and ABI 7300RT-PCR instrument (Applied Biosystems) were used to analyze quantitation levels. The amplification conditions were set as follows: 95°C for 5 minutes, 35 cycles at 95°C for 15 sec, 62°C for 35 sec, and 75°C for 30 sec. The 2^–△△CT^ method was used to quantify the expression level. The primers used were as follows: *α*-smooth muscle actin(*α*-SMA) forward: CATCACCATTGGCAACGAGC, reverse: ATCTTCATGGTGCTGGGAGC; E-cadherin forward: AGGTCTCTCTCACCACCTCC, reverse: AAATGTGTCTGGCTCCTGGG; collagen IV forward: CCTGGGCAGATTCCAAACCT, reverse: CAAAGGCGTCGTCAATCACC; fibronectin forward: ACTGGATGGGGTGGGAAT. reverse: GGAGTGGCACTGTCAACCTC; GRP78 forward: GCAGAGGGGGAGCGTTTAAT, reverse: GGTCTTCAGCTGACCTCCAC; ATF6 forward: CGAAGGGATCACCTGCTGTT, reverse: CCTGGTGTCCATCACCTGAC; *β*-actin forward: TGCTATCCCTGTACGCCTCT, reverse: TGGCCATCTCTTGCTCGAAG.

### 2.11. Western Blot

Total protein was extracted, and protein concentration was determined by the BCA method and then transferred to NC membrane (Applygen Technologies Inc. Beijing, China) after denaturation. Then, defatted milk powder was sealed at room temperature for 1 hour, and the primary antibodies were added respectively and incubated overnight at 4 °C. The primary antibodbodies used were as follows: *α*-SMA (14395-1-AP 1 : 5000), E-cadherin (20874-1-AP 1 : 1000), GRP78 (11587-1-AP 1 : 4000), ATF6 (24169-1-AP 1 : 1500), collagen IV (ab6586 1 : 2000), fibronectin (ab2413 1 : 6000). The appropriate concentration of the secondary antibody (anti-mouse RM3001 1 : 2000 or anti-rabbit SA00001-2 1 : 2000) was added. The membrane was incubated at room temperature and then washed, with *β*-actin (60008-1-1 g 1 : 4000) as control. *α*-SMA, E-cadherin, GRP78, ATF6, anti-mouse, anti-rabbit, and *β*-actin were from ProteinTech, and collagen IV and fibronectin were from Abcam. The ratio of the target protein gray value to the internal reference gray value is used to reflect the relative expression level of protein.

### 2.12. Immunofluorescence

Firstly the cells were fixed with 4% paraformaldehyde and added 0.5% Triton X-100 for 20 minutes, respectively. 5% goat serum was incubated for 1 hour at 37°C, the primary antibodies including *α*-SMA (1 : 100), E-cadherin (1 : 100), collagen IV (1 : 250), and fibronectin (1 : 100) were overnight at 4°C. FITC-labeled secondary antibody (1 : 100) was added to the cells and then incubated for 1 hour in the dark at 37°C, then DAPI was added to visualize the nucleus. Finally, cells were observed by the inverted fluorescent microscope.

### 2.13. Statistical Analysis

SPSS 21.0 was used to perform statistical analysis. Results were expressed as mean ± SD. Data obtained from each group were tested by one-way analysis of variance (ANOVA). *P* < 0.05 was considered statistically significant. *P* < 0.01 was considered greatly statistically significant.

## 3. Results

### 3.1. EGB761 Can Improve the Renal Function and Renal Tubular Injury in DKD Mice

First, we investigated whether EGB761 can improve the DKD renal function. The results showed that Scr, BUN, 24 h Pro, and FBG in the DKD group were significantly increased after 8 weeks of intragastric administration (*P* < 0.01). However, Scr, BUN, 24 h Pro, and FBG in the DKD+EGB761 group significantly decreased (*P* < 0.05, *P* < 0.01). In the DKD+4-PBA group, Scr and 24 h Pro significantly decreased (*P* < 0.05), and BUN and FBG had no significant difference. The results were shown in Table 1.

We further examined the effect of EGB761 on renal tubular injury in DKD mice. The results showed that urine *β*_2_-MG, RBP4 in the DKD group significantly increased in the DKD group (*P* < 0.01). However, urine *β*_2_-MG and RBP4 in the DKD + EGB761 group significantly decreased (*P* < 0.01), and urine RBP4 in the DKD+4-PBA group significantly decreased (*P* < 0.05), but urine *β*_2_-MG had no significant difference. Results were shown in Table 2.

### 3.2. EGB761 Can Ameliorate Renal Pathological Lesion of DKD Mice

**Figure 1:**
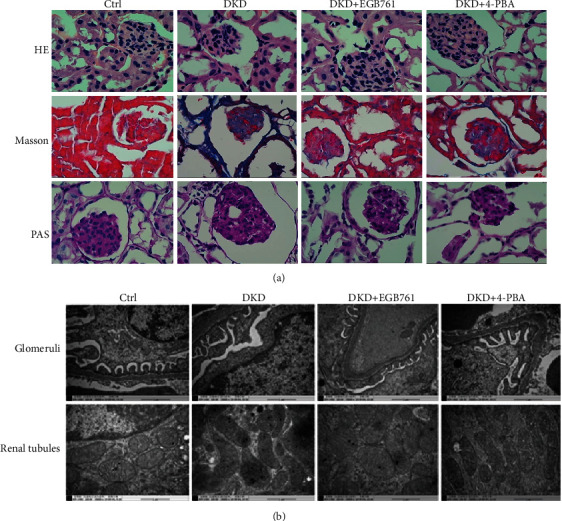
EGB761 can improve renal pathological injury in DKD mice. (a) H&E staining, Masson's staining, and PAS staining of the mice renal tissue in each group. (b) Glomeruli and renal tubule structure in transmission electron microscope in each group. Ctrl: normal group; DKD: diabetic kidney disease group; DKD + EGB761: diabetic kidney disease group+EGB761 (36 mg/kg); DKD+4-PBA: diabetic kidney disease group+4-phenylbutyrate (50 g·L-1, 1 g·kg-1).

Special kidney staining and transchromatic electron microscopy were performed to observe whether EGB761 can alleviate renal pathological lesion. Special staining of the renal tissue showed clear and complete structure of glomeruli and tubules in the Ctrl group. While DKD mice showed increased glomerular volume, collagen deposition was obvious, mesangial matrix proliferated, mesangial area widened, capillary loops increased, and renal tubular epithelial cells swelled, fused, and died. In the DKD + EGB761 group and DKD+4-PBA group, the above pathological injuries were significantly improved, as shown in Figure 1(a).

Transmission electron microscopy showed normal renal tissue structure in the Ctrl group, the thickness of the glomerular basement membrane was uniform, the podocytes were arranged orderly, and the morphology of mitochondria in the renal tubules was normal. However, in the DKD model group, the podocytes were significantly widened and fused, the glomerular basement membrane was not uniformly thickened, the mitochondria in the renal tubules were swollen, and the disappearance of the spinous fracture was observed. Compared with the DKD group, the pathological damage was ameliorated in the DKD + EGB761 group and DKD+4-PBA group, as shown in Figure 1(b).

### 3.3. EGB761 Reduced EMT in DKD

**Figure 2:**
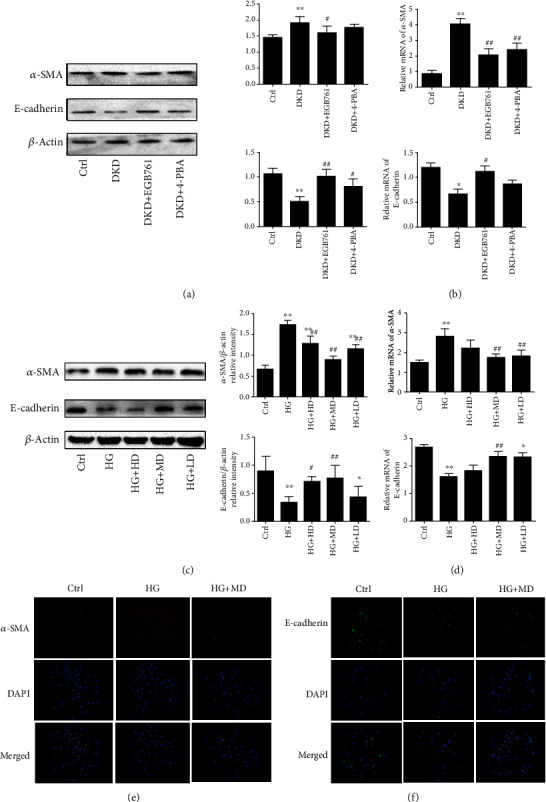
EGB761 reduced EMT in DKD. (a) The protein expression of *α*-SMA, E-cadherin, and bar graph representing the quantification of the *α*-SMA and E-cadherin bands in the renal tissue of each mice group. (b) The mRNA expression of *α*-SMA and E-cadherin in the renal tissue. (c) The protein expression of *α*-SMA, E-cadherin, and bar graph representing the quantification in HK-2 cells. (d) The mRNA expression of *α*-SMA and E-cadherin in HK-2 cells. (e) Immunofluorescent of *α*-SMA. (f) Immunofluorescent of E-cadherin. Ctrl: normal group or treated with PBS; DKD: diabetic kidney disease group; DKD + EGB761: diabetic kidney disease group+EGB761 (36 mg/kg); DKD+4-PBA: diabetic kidney disease group+4-phenylbutyrate (50 g·L-1, 1 g·kg-1); HG: high glucose; HG + HD: high glucose+EGB761 40 mg/L; HG + MD: high glucose+EGB761 20 mg/L; HG + LD: high glucose+EGB761 10 mg/L. Results were expressed as mean ± SD. ^∗^*P* < 0.05, ^∗∗^*P* < 0.01 versus Ctrl. ^#^*P* < 0.05, ^##^*P* < 0.01 versus DKD model group or HG.

We observed the effect of EGB761 on a-SMA and E-cadherin in the DKD mice kidney tissue. We found that the DKD group has a higher protein and mRNA expression of a-SMA(*P* < 0.01), while lower expression of E-cadherin (*P* < 0.01); the DKD+EGB761 has a lower protein and mRNA expression of a-SMA, while a higher expression of E-cadherin (*P* < 0.05, *P* < 0.01), which showed that EGB761 could reduce the EMT in DKD mice kidney tissues, as shown in Figures 2(a) and 2(b).

Next, we stimulated HK-2 with high glucose (30 mmol/L) to establish a cell model, intervened with different concentrations of EGB761(40 mg/L, 20 mg/L, 10 mg/L), and observed the effect of EGB761 on EMT in HK-2 through Western blot, qPCR, and cellular immunofluorescence. The results showed that the HG group had a higher protein and mRNA expression of *α*-SMA (*P* < 0.01) and lower expression of E-cadherin (*P* < 0.01). However, EGB761 could decrease the protein and mRNA expression of *α*-SMA with the most significant decrease at 20 mg/L EGB761 (*P* < 0.05, *P* < 0.01), while increases the E-cadherin expression with the most significant increase at 20 mg/L (*P* < 0.05, *P* < 0.01), as shown in Figures 2(c) and 2(d). Immunofluorescent staining showed that the DKD group has a higher expression of a-SMA, while lower expression of E-cadherin; the DKD + EGB761 has a lower expression of a-SMA, while a higher expression of E-cadherin, as shown in Figures 2(e) and 2(f).

### 3.4. EGB761 Reduced ECM Accumulation in DKD

**Figure 3:**
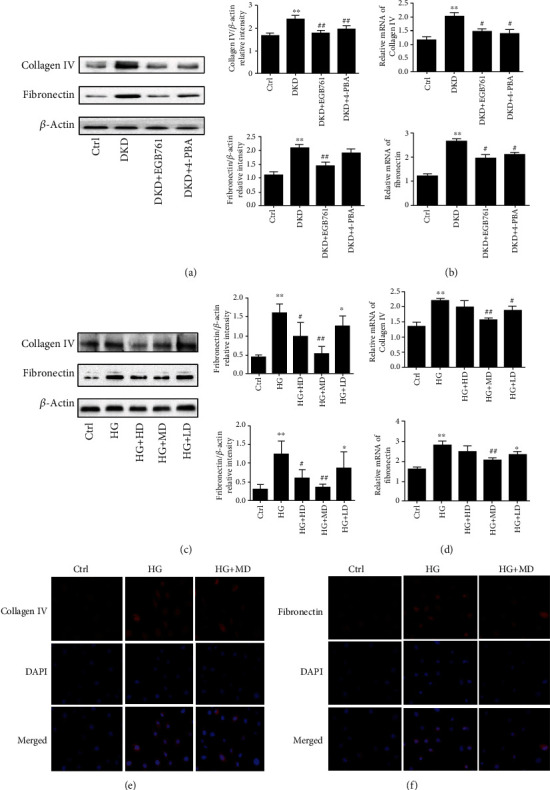
EGB761 reduced ECM accumulation in DKD. (a) The protein expression of collagen IV, fibronectin, and bar graph representing the quantification in the renal tissue. (b) The mRNA expression of collagen IV and fibronectin in the renal tissue. (c) The protein expression of collagen IV, fibronectin, and bar graph representing the quantification in HK-2 cells. (d) The mRNA expression of collagen IV and fibronectin in HK-2 cells. (e) Immunofluorescent of collagen IV. (f) Immunofluorescent of fibronectin. Ctrl: normal group or treated with PBS; DKD: diabetic kidney disease group; DKD + EGB761: diabetic kidney disease group+EGB761 (36 mg/kg); DKD+ 4-PBA: diabetic kidney disease group+4-phenylbutyrate (50 g·L-1, 1 g·kg-1); HG: high glucose; HG + HD: high glucose+EGB761 40 mg/L; HG + MD: high glucose+EGB761 20 mg/L; HG + LD: high glucose+EGB761 10 mg/L. Results are expressed as mean ± SD. ^∗^*P* < 0.05, ^∗∗^*P* < 0.01 versus Ctrl. ^#^*P* < 0.05, ^##^*P* < 0.01 versus DKD model group or HG.

We observed whether EGB761 could alleviate the ECM accumulation markers collagen IV and fibronectin in the DKD mice kidney tissue. We found that the DKD group shad a higher protein and mRNA expression of collagen IV and fibronectin (*P* < 0.01), while the DKD+EGB761 group had a lower protein and mRNA expression (*P* < 0.05, *P* < 0.01), which showed that EGB761 can reduce the ECM accumulation in DKD mice kidney tissues, as shown in Figures 3(a) and 3(b).

Next, we stimulated HK-2 with high glucose (30 mmol/L) to establish a cell model, intervened with different concentrations of EGB761 (40 mg/L, 20 mg/L, 10 mg/L), and observed the effect of EGB761 on ECM accumulation of HK-2 through Western blot, qPCR, and cellular immunofluorescence. The results showed that the HG group had a higher protein and mRNA expression of collagen IV and fibronectin (*P* < 0.01). However, EGB761 could decrease the protein and mRNA expression of collagen IV and fibronectin, with the most significant decrease at 20 mg/L (*P* < 0.05, *P* < 0.01), as shown in Figures 3(c) and 3(d). Immunofluorescent staining showed that the HG group had a higher expression of collagen IV and fibronectin, while the DKD + EGB761 group had a lower expression. The results were shown in Figures 3(e) and 3(f).

### 3.5. EGB761 Inhibited Endoplasmic Reticulum Stress in DKD

**Figure 4:**
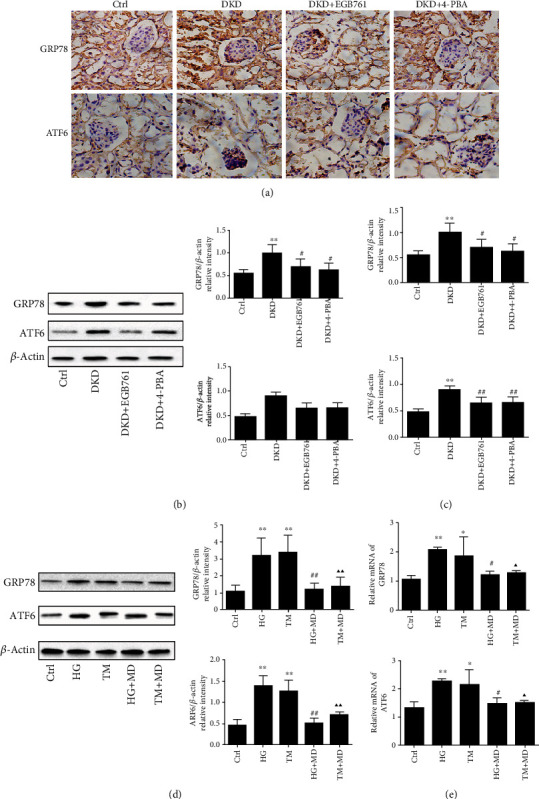
EGB761 inhibited ERS in DKD. (a) Immunohistochemical of GRP78, ATF6 in each mice group. (b) The protein expression of GRP78 and ATF6, as well as bar graph representing the quantification of the GRP78 and ATF6 bands in the renal tissue. (c) The mRNA expression of GRP78 and ATF6 in the renal tissue. (d) The protein expression of GRP78 and ATF6, as well as bar graph representing the quantification in HK-2 cells. (e) The mRNA expression of GRP78 and ATF6 in HK-2 cells. Ctrl: normal group or treated with PBS; DKD: diabetic kidney disease group; DKD + EGB761: diabetic kidney disease group+EGB761 (36 mg/kg); DKD+4-PBA: diabetic kidney disease group+4-phenylbutyrate (50 g·L-1, 1 g·kg-1); HG: high glucose; TM: tunicamycin; HG + MD: high glucose+EGB761 20 mg/L; TM + MD: tunicamycin+EGB761 20 mg/L. Results are expressed as mean ± SD. ^∗^*P* < 0.05, ^∗∗^*P* < 0.01 versus Ctrl. #*P* < 0.05, ##*P* < 0.01 versus DKD model group or HG. ▲*P* < 0.05, ▲▲*P* < 0.01 versus TM.

We observed whether EGB761 could inhibit ERS in the DKD mice kidney tissue. Immunohistochemical, Western blot, and q-PCR results showed that the DKD group had a higher expression of GRP78 and ATF6 (*P* < 0.01), while EGB761 and 4-PBA could decrease the expression (*P* < 0.05, *P* < 0.01). The above results clarified that EGB761 could relieve ERS in DKD mice, as shown in Figures 4(a)–(c).

We further took EGB761 at 20 mg/L as a safe and effective intervention concentration to explore whether EGB761 can inhibit ERS in HK-2 induced by high glucose. The HG group had a higher protein and mRNA expression of GRP78 and ATF6 (*P* < 0.01). However, both these markers were significantly decreased after the treatment with EGB761 at 20 mg/L (*P* < 0.05, *P* < 0.01), as shown in Figures 4(d) and 4(e).

### 3.6. EGB761 Attenuated the ECM Accumulation and EMT via ERS

**Figure 5:**
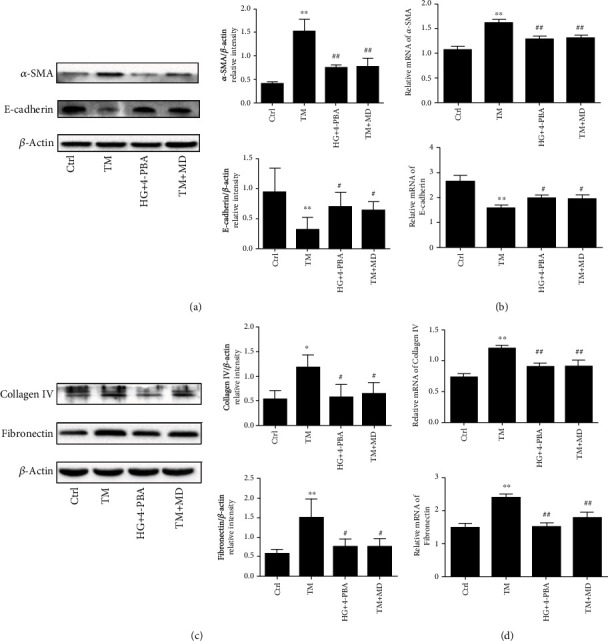
EGB761 reduced the accumulation of ECM and the expression of EMT markers in HK-2 cells via the inhibition of ERS. (a) Western blot of *α*-SMA and E-cadherin. (b) Bar graph representing the quantification of *α*-SMA, and bar graph representing the quantification of *α*-SMA and E-cadherin. (b) The mRNA expression of *α*-SMA and E-cadherin. (c) Western blot of collagen IV, fibronectin, and bar graph representing the quantification of collagen IV and fibronectin. (d) The mRNA expression of collagen IV and fibronectin. Ctrl: control treated with PBS; TM: tunicamycin; TM+4-PBA: tunicamycin+4-PBA; TM + MD: tunicamycin+EGB761 20 mg/L. Results are expressed as mean ± SD. ^∗^*P* < 0.05, ^∗∗^*P* < 0.01 versus Ctrl. #*P* < 0.05, ##*P* < 0.01 versus TM.

We further studied the mechanism of EGB761 on the ECM accumulation and EMT in HK-2. To explore whether EGB761 alleviates the ECM accumulation and EMT that were related to the inhibition of ERS, HK-2 was treated with TM (5 ug/mL),TM combined with EGB761 (20 mg/L), and TM combined with 4-PBA (1 mM) for 48 h. TM could increase the expression of *α*-SMA, collagen IV, and fibronectin (*P* < 0.05, *P* < 0.01), while decrease the expression of E-cadherin (*P* < 0.01); EGB761 and 4-PBA could decrease the expression of *α*-SMA, collagen IV, and fibronectin (*P* < 0.05, *P* < 0.01), while increase the expression of E-cadherin (*P* < 0.05), as shown in Figure 5.

## 4. Discussion

DKD is one of the major microvascular complications in diabetic patients and has become the main cause of end-stage kidney disease worldwide [1], because of the lack in effective treatment methods. In the past, it was believed that the main lesion of DKD was located in the glomerulus. In recent years, renal tubular injury in the progression of DKD was investigated. A study shows that the proportion of severe renal tubular lesion in type 2 diabetes accompanied by microalbuminuria is higher than that of glomerular lesion [13], suggesting that renal tubular lesion in DKD can predict renal function more accurately than the glomerulus. Thus, renal tubular lesion has attracted more and more attention in the pathogenesis and progression of DKD and has become a new target in DKD therapy [14].

EGB761 is an effective component in the traditional Chinese medicine. EGB761 contains flavonoids, terpene lactones, organic acids, and other chemical components, and it exerts several effects including antioxidant and free radical scavenging action, circulation improvement, platelet aggregation resistance, and nervous system protection [15]. Modern studies found that Ginkgo biloba injection significantly reduces 24-hour urinary microalbumin, total cholesterol, and triglyceride and improves renal function [16, 17] in DKD patients, as well as relieves their clinical symptoms. However, its specific role and mechanism are needed to be clarified.

Our research shows that EGB761 can improve 24 h Pro, Scr, BUN, and FBG in DKD mice. By kidney tissue-specific staining and transmission electron microscopy, we confirmed that EGB761 can improve the pathological damage of glomerulus and renal tubules. In order to further explore whether EGB761 can improve DKD renal tubular damage, the urine *β*_2_-MG and RBP4 levels were tested by ELLSA experiment, and the results shows that EGB761 could decrease the levels of *β*_2_ -MG and RBP4; so, the therapeutic effect of EGB761 on DKD was confirmed in vivo experiments.

One of the important pathological features of renal tubular injury is renal interstitial fibrosis, which mainly manifested as ECM deposition [18]. When the kidney function is impaired at an early stage, locally infiltrated inflammatory cells release many soluble cytokines, growth factors, and vasoactive substances, which promote plasma proteins, including fibrinogen and fibronectin, to penetrate into the damaged site and form ECM deposition. This ECM can attach fibroblasts and immune cells to the damaged parts to promote repair or pathogen removal [19]. If the damage intensity is too high or persists, ECM is hydrolyzed into small fragments with biological activity, activating surrounding cells and producing fibrotic ECM such as collagen and fibronectin, which are not easy to degrade. The renal tissue structure is continuously compressed until it disappears. More and more collagen and fibronectin are deposited to form dense and firm ECM, which leads to changes in the matrix structure and function and matrix hardening affecting signal transmission, thus forming interstitial fibrosis [20].

EMT is also one of the important causes of renal tubulointerstitial fibrosis in DKD. EMT enables polarized epithelial cells to have mesenchymal cell phenotype through various biochemical changes [21]. After conversion, inflammatory cells were attracted, myofibroblasts activated, causing ECM abnormal generation [22], further aggravating renal tubulointerstitial fibrosis.

Our study showed that EGB761 could decrease the expression of ECM deposition and EMT markers. In HK-2 cells, high glucose promoted ECM deposition and EMT while EGB761 could effectively reduce the ECM accumulation and EMT in HK-2 cells. Therefore, EGB761 especially at the concentration of 20 mg/L could be considered as a safe and effective concentration.

ERS is involved in the pathogenesis of DKD and plays an important role in the development of renal fibrosis in DKD [23]. Activated transcription factor 6 (ATF6) is one of the major transcription proteins on the endoplasmic reticulum, which binds to its partner glucose regulatory protein 78 (GRP78) in an inactive state under nonstress stress; however, stressed state occurs, and it dissolves from it, triggering unfolded protein response (UPR) [24]. UPR protects cells by inhibiting endoplasmic reticulum overload by suspending protein synthesis, promoting protein degradation, and upregulating the expression of ERS proteins [25]. However, if stress persists or is too strong, the endoplasmic reticulum stress-related apoptosis pathway is activated, and cell apoptosis is induced [26]. A previous study by our research group found that ERS participates in ECM accumulation and EMT in early type 1 diabetic mice and HK-2 cells, while ERS inhibitor 4-PBA can block the occurrence of EMT markers that increased expression and ECM accumulation [27]. Moon et al. also found that ER stress agonist tunicamycin induces EMT in HK2 cells, while ERS inhibitor 4-PBA weakens EMT in HK-2 cells induced by tunicamycin [28]. However, whether EGB761 can reduce the ECM accumulation and EMT in HK-2 cells through ERS has not been reported yet.

Immunohistochemistry, Western blot, and q-PCR showed that EGB761 could reduce the expression of GRP78 and ATF6 in DKD mice. In HK-2 cells, we also found that EGB761 could decrease the protein and mRNA expression of GRP78 and ATF6, indicating that EGB761 could inhibit the endoplasmic reticulum stress state of DKD. However, whether EGB761 can reduce the ECM accumulation and EMT of DKD by inhibiting endoplasmic reticulum stress is unclear. Therefore, the mechanism of EGB761 in reducing the accumulation of ECM and EMT was further explored in HK-2 cells, using tunicamycin, an endoplasmic reticulum stressor, and 4-PBA, an inhibitor of endoplasmic reticulum stress. Our results demonstrated that EGB761 could improve both the accumulation of ECM and the EMT, partly by inhibiting ERS.

## 5. Conclusion

EGB761 might improve DKD by inhibiting ECM accumulation, expression of EMT markers, and endoplasmic reticulum stress. However, EGB761 is composed of various chemical components, and its effective component against DKD might be one or more than one. Thus, the precise active principle exerting the effect of improving DKD by inhibiting endoplasmic reticulum stress remains to be further clarified, and it will be the aim of our next study.

## Figures and Tables

**Table 1:** Detection results of 24 h Pro, BUN, Scr contents, and fasting glucose level of mice in each group (*x* ± *s*, *n* = 10).

	24 h Pro/ (mg·24 h^−1^)	Scr/ (*μ*mol·L^−1^)	BUN/ (mmol·L^−1^)	FBG/ (mmol·L^−1^)
Ctrl	6.61 ± 1.78	8.71 ± 1.80	7.99 ± 2.67	6.89 ± 1.17
DKD	17.16 ± 3.11^∗∗^	27.72 ± 4.84^∗∗^	24.42 ± 6.64^∗∗^	22.59 ± 3.87^∗∗^
DKD+EGB761	10.79 ± 2.27^##^	20.96 ± 4.51^#^	16.69 ± 4.62^#^	16.47 ± 5.12^#^
DKD+4-PBA	12.81 ± 1.98^#^	19.97 ± 5.35^#^	18.86 ± 6.29	22.77 ± 2.05

Notes: results are expressed as mean ± SD. ^∗∗^*P* < 0.01 versus Ctrl. #*P* < 0.05, ##*P* < 0.01 versus DKD group.

**Table 2:** Urine *β*_2_-MG and RBP4 levels of mice in each group(^−^*x* ± *s*, *n* = 10).

	*β*2-MG (mg·L^−1^)	RBP4/ (mg·L^−1^)
Ctrl	6.17 ± 1.75	10.96 ± 2.24
DKD	34.54 ± 11.76^∗∗^	32.24 ± 9.72^∗∗^
DKD + EGB761	18.93 ± 6.06^##^	14.51 ± 2.96^##^
DKD+4-PBA	25.44 ± 7.98	20.22 ± 4.86^#^

Notes: results are expressed as mean ± SD. ^∗∗^*P* < 0.01 versus Ctrl. #*P* < 0.05, ##*P* < 0.01 versus DKD group.

## Data Availability

The data used to support the findings of this study are available from the corresponding author upon request.
